# Anatomic distribution of lower extremity deep venous thrombosis is associated with an increased risk of pulmonary embolism: A 10-year retrospective analysis

**DOI:** 10.3389/fcvm.2023.1154875

**Published:** 2023-03-22

**Authors:** Jianjun Zhang, Yan Chen, Zhicong Wang, Xi Chen, Yuehong Liu, Mozhen Liu

**Affiliations:** ^1^ Department of Orthopedics, People’s Hospital of Deyang City, Deyang, China; ^2^ Department of Orthopedics, The First Affiliated Hospital of Dalian Medical University, Dalian, China

**Keywords:** pulmonary embolism, lower extremity deep venous thrombosis, anatomic distribution, risk factor, compression ultrasonography

## Abstract

**Aims:**

To investigate the potential relationship between anatomic distribution of lower extremity deep venous thrombosis (LEDVT) and pulmonary embolism (PE).

**Methods:**

A retrospective case-control study was performed in patients diagnosed with LEDVT, which were confirmed by bilateral lower extremity compression ultrasonography (CUS) examination. According to the ultrasound reports, thrombus sidedness was categorized as unilateral and bilateral lower extremity, thrombus location was classified into distal and proximal LEDVT. Anatomic distributions of LEDVT were further subdivided depending on the combination of thrombus sidedness and location. Patients with PE were identified using the International Classification of Diseases-10 (ICD-10) codes (I26.0 and I26.9), and divided into PE group and Non-PE group. Univariate and multivariate logistic regression analyses were used to assess the association between anatomic distribution of LEDVT and PE. Sensitivity analyses were also conducted.

**Results:**

A total of 2,363 consecutive patients with LEDVT were included, of whom 66.10% and 33.90% were unilateral and bilateral LEDVT, as well as 71.39% and 28.61% were isolated distal and proximal LEDVT, respectively. After the diagnosis of LEDVT, 185 patients (7.83%) developed PE. The proportions of PE ranged between the lowest (4.07%) in unilateral-distal LEDVT and highest (14.55%) in bilateral-proximal LEDVT. Multivariate logistic regression analysis showed that bilateral LEDVT (odds ratios [OR] = 2.455, 95% confidence interval [CI]: 1.803–3.344, *P* < 0.001) and proximal LEDVT (OR = 1.530, 95% CI: 1.105–2.118, *P* = 0.010) were risk factors for developing PE. Moreover, unilateral-proximal (OR = 2.129, 95% CI: 1.365–3.320, *P* = 0.00), bilateral-distal (OR = 3.193, 95% CI: 2.146–4.752, *P* < 0.001) and bilateral-proximal LEDVT(OR = 3.425, 95% CI: 2.093–5.603, *P* < 0.001) were significantly associated with an increased risk of PE. Sensitivity analyses also confirmed the robustness of these associations.

**Conclusion:**

Patients with unilateral-proximal, bilateral-distal or bilateral-proximal are more likely to suffer from PE than those with unilateral-distal LEDVT.

## Introduction

Deep venous thrombosis (DVT), the formation of blood thrombus in the deep veins, remains a serious and growing public health problem (1). In several large population-based studies, the overall incidence rates of DVT per 100,000 person-years were as high as 147 in USA (2), 123 in Taiwan (3), 108 in Norway (4), and 80.9 in Canada (5). As is well known, thrombus originating in the lower extremity into the pulmonary arteries is considered to be the most common mechanism for pulmonary embolism (PE) (6). With the increasing number of hospitalized DVT patients, the incidence rate of PE was also markedly elevated (7–9). Notably, PE has become the third leading cause of cardiovascular death globally, accounting for 8–13 per 1,000 deaths in women and 2–7 per 1,000 deaths in men (10). On the other hand, global public awareness for DVT and PE was significantly lower than other thromboembolic disease (11). To date, venous thromboembolism (VTE), including DVT and PE, continues to impose a substantial social and economic burden worldwide (11).

With regard to DVT treatment, one of the most important goals is to detect and prevent the occurrence of PE (12). Due to the lack of specific clinical symptoms and signs, diagnosing PE remains a clinical challenge (13). Computed tomography pulmonary angiography (CTPA), which requires the injection of iodinated contrast material, has been widely used as a gold-standard diagnostic modality in patients with suspected PE (14). However, CTPA exposes patients to risks of false-positive result, allergic reaction, renal failure and cumulative radiation-induced cancer (13, 14). Moreover, CTPA was found to be overused, leading to ineffective utilization of hospital resources (15). Therefore, there is an urgent need to identify DVT patients at high risk of PE, and to implement effective preventive measures (12).

Recently, the occurrence of PE seems to be closely related to the anatomic distribution of lower extremity DVT (LEDVT) (16, 17). In general, isolated distal LEDVT is presumed to be more benign than proximal LEDVT, presenting a lower risk of PE, VTE recurrence, post-thrombotic syndrome and mortality (18–20). On the other hand, thrombus sidedness, especially bilateral LEDVT, was found to be associated with an increased risk of PE (12, 17). However, limited studies have focused on the single relationship between thrombus sidedness or thrombus location and PE, and yielded inconsistent results (12, 16, 17, 21–24).

Hospital information system (HIS) is one of the most widely used information systems in the health care. In this study, we used our hospital HIS database over a 10-year period (2012–2022) to identify all LEDVT patients, and then explored the association between thrombus sidedness, thrombus location and risk of PE. Subsequently, anatomic distributions of LEDVT were subdivided depending on the combination of thrombus sidedness and location, and further analyzed this relationship in different anatomic distribution of LEDVT.

## Materials and methods

### Study subjects and design

**Figure 1:**
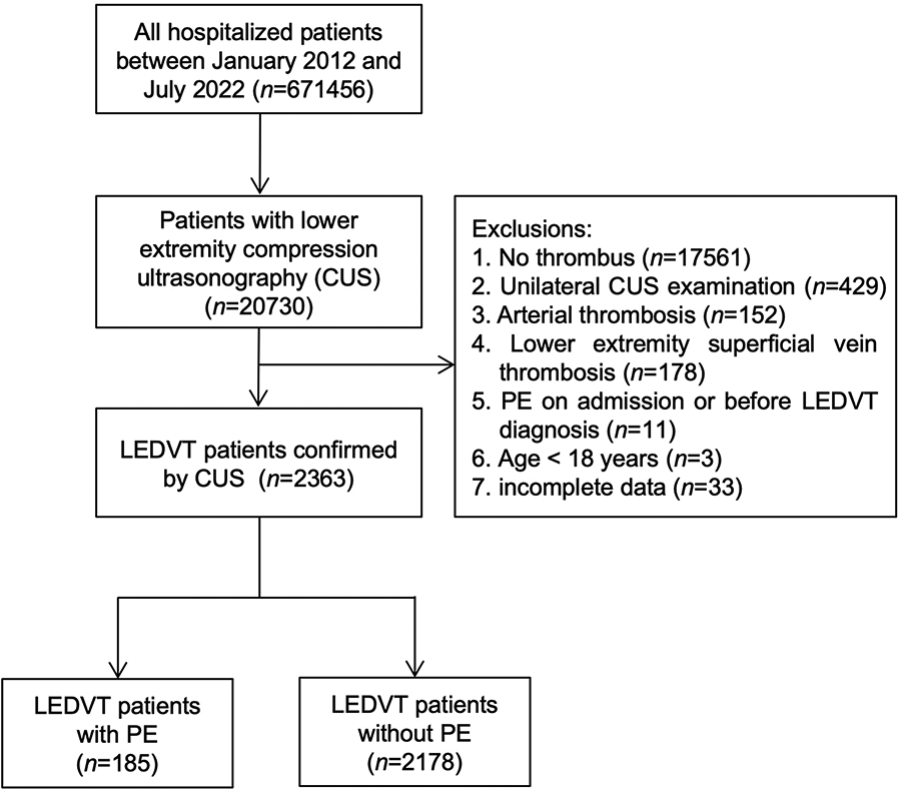
Flowchart of patient inclusion and exclusion. CUS, compression ultrasonography; PE, pulmonary embolism; LEDVT, lower extremity deep venous thrombosis.

This retrospective case-control study was conducted at People's Hospital of Deyang City, a 1838-bed tertiary hospital located in Southwest China. Between January 1, 2012 and July 31, 2022, all hospitalized patients were screened by searching the electronic HIS database (*n* = 671,456). Initially, 20,730 patients who underwent lower extremity compression ultrasonography (CUS) were identified. After that, patients were excluded if they met any of the following criteria: (1) no thrombus; (2) unilateral CUS examination; (3) arterial thrombosis; (4) lower extremity superficial vein thrombosis; (5) PE on admission or before LEDVT diagnosis; (6) age <18 years; (7) incomplete data. The study flowchart is illustrated in Figure 1. The study protocol was approved by the Institutional Ethics Committee of our hospital (Number: 2021-04-019-K01), and performed in accordance with the Declaration of Helsinki. Patient informed consent was waived owing to the use of anonymous retrospective data.

### Data collection and definition

For diagnosis of LEDVT, CUS examinations were generally performed by an experienced radiologist, and the results were reviewed and verified by another radiologist. Briefly, the following deep veins of the thigh and calf were sequentially scanned: common femoral vein, superficial femoral vein, deep femoral vein, popliteal vein, anterior tibial vein, posterior tibial vein, peroneal vein and calf muscle vein. According to the results of ultrasound reports, thrombus sidedness included left, right and bilateral lower extremity, and thrombus location included proximal (thrombus occurring in the popliteal vein and/or above), distal (thrombus below the popliteal vein), and mixed LEDVT (both proximal and distal thrombus) (23). Previous studies have found a similar PE risk between left and right LEDVT (12, 16, 24), and this study also did not reach statistical significance (Supplementary Table S1 and Figure S1). For this reason, thrombus sidedness was categorized as unilateral or bilateral lower extremity. Since LEDVT is thought to progress from distal to proximal location, and the number of isolated proximal LEDVT patients was relatively small in the study, subjects with both distal and proximal LEDVT were regarded as proximal LEDVT (25). Thereafter, anatomic distributions of LEDVT were further subdivided into 4 groups: unilateral-distal, unilateral-proximal, bilateral-distal and bilateral-proximal LEDVT.

At our institute, the diagnosis of PE was based on the presence of an intraluminal filling defect in the pulmonary artery tree on CTPA. In accordance with a previous study (26), the International Classification of Diseases-10 (ICD-10) codes were used to identify patients with PE (I26.0 and I26.9). ICD-10 I26.0 represents PE patients with acute cor pulmonale, while I26.9 represents PE patients without acute cor pulmonale. Meanwhile, the diagnosis of PE was confirmed using discharge diagnoses as “pulmonary embolism, pulmonary thrombosis, pulmonary artery embolism or pulmonary infarction”. Thereby, patients with LEDVT were divided into PE group (case subjects with PE) and Non-PE group (control subjects without PE).

Moreover, other clinical characteristics were collected, including age at admission, sex, body mass index (BMI), as well as history of tobacco and alcohol use which were recorded in the electronic admission note. Based on the Working Group on Obesity in China (27), obesity was defined as BMI ≥ 28.0 kg/m^2^. Smoking and drinking status were classified as never, former, current or unknown. Also, comorbidities associated with PE were extracted using ICD-10 codes of discharge diagnoses (28): hypertension (I10–I13, I15), diabetes mellitus (E11–E14), chronic obstructive pulmonary disease (COPD, J42–J44), atrial fibrillation (I48), heart failure (I50), varicose vein (I83.9), hepatic insufficiency (K72.0–K72.1, K72.9), renal insufficiency (N17–N19), and cancer (C00–C97, D00–D09). In this study, all data were cleaned independently by two authors (JZ and YC), and cross-checked for accuracy.

### Statistical analysis

Prior to analysis, all variables were checked for missing values, and 19.85% of obesity data were found to be missing (*n* = 469). Considering the large number of missing value, we created a missing obesity category using missing indicator method rather than multiple imputation (29).

For continuous variable (age), normality was first checked using the Shapiro-Wilk test, and described as mean ± standard deviation (SD), whereas other categorical variables were reported as numbers (percentages). Differences between the two groups were compared by Student's *t*-test for continuous variable and Pearson's *χ*^2^ test for categorical variables. The proportions of PE with 95% confidence intervals (CI) were calculated using the Wilson/Brown method. Differences in proportions were evaluated by *χ*^2^ test, and *P* values were adjusted for multiple comparisons using the Benjamini-Hochberg false discovery rate (FDR) correction, with the FDR set at 5% (30).

Univariate logistic regression analyses were performed to identify factors associated with PE. All variables with *P* value ≤0.1 in univariate analyses were included in the multivariate logistic regression analyses. The assumption of linearity in the logit for the continuous variable was checked by the Box-Tidwell test, it was found to be violated for age. For this, age was transformed into categorical variable based on the cut-off value determined by the receiver operating characteristic curve (ROC) analysis. Multicollinearity was evaluated by variance inflation factor (VIF), with a VIF >10 indicating excessive multicollinearity (31). In this study, two multivariate analysis models were constructed. Model 1 was adjusted for thrombus sidedness (unilateral/bilateral), thrombus location (distal/proximal), and statistically significant variables (age, sex, obesity, hypertension, renal insufficiency, cancer), while Model 2 was adjusted for anatomic distribution of LEDVT (unilateral-distal/unilateral-proximal/bilateral-distal/bilateral-proximal) and statistically significant variables. Moreover, sensitivity analyses excluding those patients with missing data were conducted to test the robustness of the results. Using logistic regression analyses, crude and adjusted odds ratios (OR) with 95% CI were calculated. Existing studies have shown that patients with unilateral or distal LEDVT were less likely to suffer from PE (12, 23). Thus, unilateral, distal and unilateral-distal LEDVT was used as a reference.

All reported *P* values are two-sided, and *P* < 0.05 was considered statistically significant. All analyses were conducted using JMP Pro software (version 16.0.0; SAS Institute Inc., Cary, NC, United States) and GraphPad Prism (version 9.1.1; GraphPad Software, San Diego, California, United States).

## Results

### Patient characteristics

As confirmed by ultrasound, a total of 2,363 consecutive hospitalized patients with LEDVT were included in the final analysis. Patient characteristics are summarized in Table 1. The mean age was 67.61 years (ranging from 20 to 98 years), and 51.54% were women. Among these patients, 801 (33.90%) were bilateral LEDVT, and 1,562 (66.10%) were unilateral LEDVT (852 in the left and 710 in the right lower extremity). For the thrombus location, 1,687 (71.39%) had isolated distal LEDVT, 676 (28.61%) had proximal LEDVT (417 with distal LEDVT and 259 without distal LEDVT). During hospitalization, 185 patients developed PE after the diagnosis of LEDVT. Overall, the proportion of PE among LEDVT patients was 7.83% (95% CI: 6.81%–8.98%). More specifically, the proportions of PE were 5.57% (95% CI: 4.54%–6.82%) for unilateral LEDVT, and 12.24% (95% CI: 10.14%–14.69%) for bilateral LEDVT, the difference was statistically significant (*P* < 0.001). Regarding the thrombus location, patients with proximal LEDVT (10.95%, 95% CI: 8.81%–13.52%) had a higher proportion of PE than those with distal LEDVT (6.58%, 95% CI: 5.49%–7.86%, *P* < 0.001). When compared with patients without PE, patients with PE were older and obesity (*P* < 0.05). The other characteristics were comparable between the two groups, except for hypertension, renal insufficiency and cancer (*P* < 0.05).

**Table 1:** Patient characteristics.

Variables	All patients (*n* = 2,363)	PE group (*n* = 185)	Non-PE group (*n* = 2,178)	*P* value
Age, years (mean ± SD)	67.61 ± 12.20	69.78 ± 13.16	67.08 ± 11.76	0.001
Sex, *n* (%)				0.082
Men	1,145 (48.46)	101 (54.59)	1,044 (47.93)	
Women	1,218 (51.54)	84 (45.41)	1,134 (52.07)	
Obesity, *n* (%)	172 (7.28)	19 (10.27)	153 (7.02)	0.014
Smoking status, *n* (%)				0.303
Never smoker	1,597 (67.58)	122 (65.95)	1,475 (67.72)	
Current smoker	414 (17.52)	28 (15.14)	386 (17.72)	
Former smoker	252 (10.66)	27 (14.59)	225 (10.33)	
Unknown	100 (4.23)	8 (4.32)	92 (4.22)	
Drinking status, *n* (%)				0.511
Never drinker	1,310 (55.44)	102 (55.14)	1,208 (55.46)	
Current drinker	547 (23.15)	37 (20.00)	510 (23.42)	
Former drinker	137 (5.80)	14 (7.57)	123 (5.65)	
Unknown	369 (15.62)	32 (17.30)	337 (15.47)	
Comorbidities, *n* (%)				
Hypertension	896 (37.92)	51 (27.57)	845 (38.80)	0.003
Diabetes mellitus	495 (20.95)	33 (17.84)	462 (21.21)	0.279
COPD	453 (19.17)	38 (20.54)	415 (19.05)	0.622
Atrial fibrillation	217 (9.18)	21 (11.35)	196 (9.00)	0.288
Heart failure	103 (4.36)	7 (3.78)	96 (4.41)	0.690
Varicose vein	100 (4.23)	8 (4.32)	92 (4.22)	0.948
Hepatic insufficiency	126 (5.33)	11 (5.95)	115 (5.28)	0.699
Renal insufficiency	195 (8.25)	8 (4.32)	187 (8.59)	0.043
Cancer	352 (14.90)	38 (20.54)	314 (14.42)	0.025
Thrombus sidedness, *n* (%)				<0.001
Unilateral LEDVT	1,562 (66.10)	87 (47.03)	1,475 (67.72)	
Bilateral LEDVT	801 (33.90)	98 (52.97)	703 (32.28)	
Thrombus location, *n* (%)				<0.001
Distal LEDVT	1,687 (71.39)	111 (60.00)	1,576 (72.36)	
Proximal LEDVT	676 (28.61)	74 (40.00)	602 (27.64)	

PE, pulmonary embolism; SD, standard deviation; COPD, chronic obstructive pulmonary disease; LEDVT, lower extremity deep venous thrombosis.

### Anatomic distribution of LEDVT

As shown in Table 2, unilateral-distal LEDVT (46.08%) was the most common type of thrombosis, whereas PE occurred most frequently in bilateral-distal LEDVT (35.68%). Based on the anatomic distribution of LEDVT, the proportions of PE ranged between the lowest (4.07%, 95% CI: 3.05%–5.40%) in unilateral-distal LEDVT and highest (14.55%, 95% CI: 10.50%–19.81%) in bilateral-proximal LEDVT. When compared with unilateral-distal LEDVT, patients with unilateral-proximal, bilateral-distal and bilateral-proximal LEDVT had higher proportions of PE (adjusted *P* < 0.001).

**Table 2:** Proportions of pulmonary embolism stratified by the anatomic distribution of lower extremity deep venous thrombosis.

	All patients, *n* (%)	PE patients, *n* (%)	Proportions (%, 95% CI)	Unadjusted *P* value^a^	Adjusted *P* value^b^
Unilateral LEDVT					
Distal	1,106 (46.08)	45 (24.32)	4.07 (3.05–5.40)	–	–
Proximal	456 (19.30)	42 (22.70)	9.21 (6.89–12.22)	5.6 × 10^−5^	5.9 × 10^−5^
Bilateral LEDVT					
Distal	581 (24.59)	66 (35.68)	11.36 (9.03–14.20)	9.5 × 10^−9^	1.5 × 10^−8^
Proximal	220 (9.31)	32 (17.30)	14.55 (10.50–19.81)	1.3 × 10^−9^	4.1 × 10^−9^

LEDVT, lower extremity deep venous thrombosis; PE, pulmonary embolism; CI, confidence interval.

^a^ *P* values were obtained by *χ*^2^ test as compared to the reference category (unilateral-distal LEDVT).

^b^ *P* values were adjusted for multiple comparisons using the Benjamini-Hochberg false discovery rate correction.

### Thrombus sidedness and location associated with PE

Using ROC analysis for PE risk (Supplementary Figure S2), the optimal cut-off point for age was 75.0 years [area under curve (AUC): 0.575, 95% CI: 0.536–0.614; sensitivity: 77.30%, specificity: 38.02%]. As shown in Table 3, univariate analyses found that age, obesity, hypertension, renal insufficiency, cancer, thrombus sidedness and thrombus location were significantly associated with PE (*P* < 0.05). Sex was close to achieving statistical significance (*P* = 0.083). The above factors were entered into the multivariate logistic analysis (Model 1), and multicollinearity results showed that VIF ranged from 1.014 to 1.762 (Supplementary Table S2), indicating that there was no multicollinearity between these variables. In the multivariate analysis, obesity, hypertension, thrombus sidedness and thrombus location were independently associated with PE (*P* < 0.05). In other words, bilateral LEDVT (OR = 2.455, 95% CI: 1.803–3.344, *P* < 0.001) and proximal LEDVT (OR = 1.530, 95% CI: 1.105–2.118, *P* = 0.010) were risk factors for developing PE.

**Table 3:** Univariate and multivariate logistic regression analyses of factors associated with pulmonary embolism (Model 1).

Variables	Univariate		Multivariate (Model 1)	
	OR (95% CI)	*P* value	OR (95% CI)	*P* value
Age (≥75 vs. <75 years)	1.673 (1.205–2.323)	0.002	1.410 (0.998–1.992)	0.051
Sex (men vs. women)	1.306 (0.966–1.765)	0.083	1.296 (0.950–1.770)	0.102
Obesity (yes vs. no)	1.688 (1.012–2.817)	0.045	1.810 (1.068–3.066)	0.027
Hypertension (yes vs. no)	0.600 (0.430–0.838)	0.003	0.693 (0.488–0.984)	0.041
Renal insufficiency (yes vs. no)	0.481 (0.233–0.993)	0.048	0.542 (0.258–1.138)	0.105
Cancer (yes vs. no)	1.535 (1.053–2.235)	0.026	1.199 (0.808–1.780)	0.366
Thrombus sidedness (vs. unilateral)				
Bilateral LEDVT	2.363 (1.747–3.198)	<0.001	2.455 (1.803–3.344)	<0.001
Thrombus location (vs. distal)				
Proximal LEDVT	1.745 (1.282–2.377)	<0.001	1.530 (1.105–2.118)	0.010

OR, odds ratio; CI, confidence interval; LEDVT, lower extremity deep venous thrombosis.

### Anatomic distribution of LEDVT associated with PE

Anatomic distribution of LEDVT, instead of thrombus sidedness and thrombus location, was included into the multivariate logistic analysis (Model 2, Table 4), and no significant multicollinearity was detected between variables (Supplementary Table S2). When compared with unilateral-distal LEDVT, the adjusted OR for PE was highest in bilateral-proximal LEDVT (OR = 3.425, *P* < 0.001), followed by bilateral-distal LEDVT (OR = 3.193, *P* < 0.001) and unilateral-proximal LEDVT (OR = 2.129, *P* = 0.001). In addition, obesity and hypertension were also found to be independently associated with PE (*P* < 0.05).

**Table 4:** Multivariate logistic regression analyses of factors associated with pulmonary embolism (Model 2).

Variables	OR (95% CI)	*P* value
Age (≥75 vs. <75 years)	1.409 (0.997–1.992)	0.052
Sex (men vs. women)	1.328 (0.972–1.814)	0.075
Obesity (yes vs. no)	1.808 (1.067–3.064)	0.028
Hypertension (yes vs. no)	0.687 (0.484–0.976)	0.036
Renal insufficiency (yes vs. no)	0.542 (0.258–1.139)	0.106
Cancer (yes vs. no)	1.243 (0.837–1.847)	0.281
Anatomic distribution of LEDVT (vs. unilateral-distal)		
Unilateral-proximal	2.129 (1.365–3.320)	0.001
Bilateral-distal	3.193 (2.146–4.752)	<0.001
Bilateral-proximal	3.425 (2.093–5.603)	<0.001

OR, odds ratio; CI, confidence interval; LEDVT, lower extremity deep venous thrombosis.

### Sensitivity analysis

**Figure 2:**
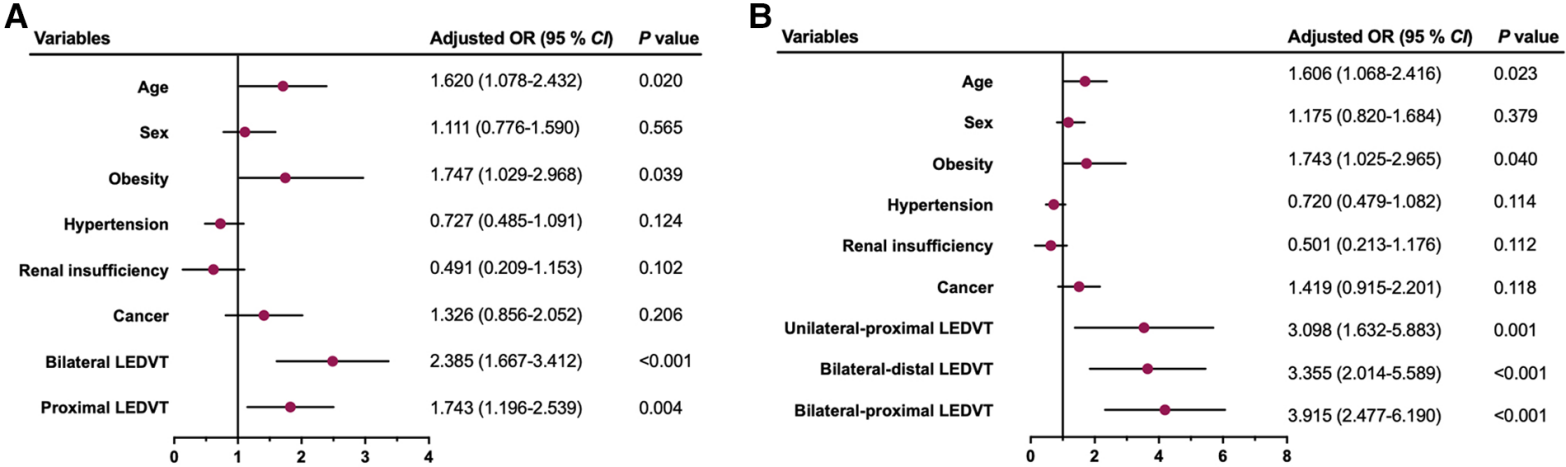
Multivariate sensitivity analysis by excluding patients with missing data for the risk of pulmonary embolism. (**A**) Model 1. (**B**) Model 2. OR, odds ratio; CI, confidence interval; LEDVT, lower extremity deep venous thrombosis.

After excluding 469 patients with missing data, 1,894 patients were assessed for sensitivity analyses. The results are graphically illustrated as forest plots in Figure 2. Consistent with the main analyses, bilateral and proximal LEDVT remained independently associated with an increased risk of PE (Model 1, *P* < 0.01, Figure 2A), whereas unilateral-proximal, bilateral-distal and bilateral-proximal LEDVT exhibited higher risk of PE (Model 2, *P* < 0.01, Figure 2B).

## Discussion

Although DVT and PE often differ substantially in terms of risk factors, disease presentation and clinical outcomes, DVT is believed to contribute to the occurrence of PE (32). Recently, data from the RE-COVERY DVT/PE global observational study showed that 35.21% of PE patients had LEDVT, while 20.63% of LEDVT patients had PE (33). In another international, prospective, observational WHITE study, 10.21% of LEDVT patients had confirmed concomitant PE (34). After the diagnosis of LEDVT, we found the proportion of PE among LEDVT patients was 7.83%, which was lower than the above-mentioned (33, 34), but slightly higher than that reported in another study (6.15%) (16).

CUS is the first-line tool for imaging LEDVT, and recommended to undergo bilateral examinations because of a high incidence of clinically silent contralateral thrombosis (35). Using the ultrasound, anatomic distribution of LEDVT can be observed clearly, including thrombus sidedness (left, right and bilateral) and thrombus location (proximal, distal and mixed). In the present study, patients with bilateral LEDVT had a higher proportion of PE than those with unilateral LEDVT (12.24% vs. 5.57%). This association remained significant even after multivariable adjustments and sensitivity analysis (Model 1). This finding indicated that bilateral LEDVT was independent risk factor for developing PE, leading to a 2.455-fold increase in PE risk. Consistent with our study, Bikdeli et al. (17) included 30,445 patients with LEDVT, and patients with bilateral DVT had markedly higher rates of PE than left or right LEDVT (46.62% vs. 22.13% vs. 24.83%), as well as a significantly higher rate of subsequent 90-day new PE (1.73% vs. 0.72% vs. 0.91%). This relationship was also confirmed by other studies (16, 24).

In addition, several prior studies have shown that patients with right LEDVT were more likely to suffer from PE than those with left LEDVT (12, 23, 36). Iliac vein compression syndrome, also known as May-Thurner syndrome, is an uncommon anatomical variant characterized by compression of left common iliac vein by the overlying right iliac artery. For this, left iliac vein compression may potentially limit the migration of the thrombus from this stenotic segment to the pulmonary arteries, leading to a relatively low incidence of PE (36). This speculation was supported by the results of Chen et al. (36), who confirmed that left iliac vein thrombosis (IVT) was associated with a lower incidence of symptomatic PE than right LEDVT (5.4% vs. 13.8%). In this study, we observed a similar trend, but this was not statistically significant in multivariate analysis (Supplementary Table S1, *P* = 0.053) and sensitivity analysis (Supplementary Figure S1, *P* = 0.113). Also, the literature mentioned above reported no differences in PE incidence between left non-IVT, right IVT and right non-IVT (12.8% vs. 10.1% vs. 16.6%, *P* = 0.38) (36). Due to a relatively small number of proximal LEDVT patients included in this study, this may be the reason that right LEDVT did not reach a statistically significant level.

Commonly, patients with proximal LEDVT had a higher likelihood for development of PE than those with distal LEDVT (18, 23). For this, the CHEST guideline emphasizes the importance of thrombus location in treatment choices between serial imaging and anticoagulation therapy (37). As recommended by the 2019 European Society of Cardiology (ESC) guideline, thrombus location can also be used to confirm PE (38). Except for the different anatomic location, proximal and distal LEDVT differ substantially in many aspects, including age, sex, comorbidity burden and VTE risk factors (e.g., recent surgery) (18, 19, 33). After adjusting for confounding factors, we also found that proximal LEDVT was risk factor for developing PE in multivariate analysis and sensitivity analysis (Model 1).

Based on the results of thrombus sidedness and thrombus location, we further subdivided the anatomic distributions of LEDVT into 4 categories (unilateral-distal, unilateral-proximal, bilateral-distal and bilateral-proximal LEDVT). In this study, the proportions of PE ranged between the lowest (4.07%) in unilateral-distal LEDVT and highest (14.55%) in bilateral-proximal LEDVT. The results from multivariate analysis also found that unilateral-proximal, bilateral-distal and bilateral-proximal LEDVT exhibited a 2.129-fold, 3.193-fold and 3.425-fold increase risk for PE, respectively (Model 2). In line with this, Qiu et al. (16) reported that the incidence of PE was highest in patients with bilateral-proximal LEDVT (15.4%), followed by bilateral-distal LEDVT (11.1%), left-proximal LEDVT (7.2%) and right-proximal LEDVT (5.5%). In fact, the clinicians have paid more attention to patients with proximal LEDVT, as evidenced by the treating decision of extension of secondary prophylaxis (34). Notably, the PE risk in isolated distal LEDVT was still high, even without leg edema and/or pain (39). As bilateral-distal LEDVT (24.59%) was the second most prevalent thrombosis after unilateral-distal LEDVT in the study, anatomic distributions of LEDVT deserved special attention, especially bilateral-distal LEDVT.

Moreover, obesity and hypertension were found to be independently associated with PE, while age (Model 2, *P* = 0.052) and sex (Model 2, *P* = 0.075) were close to achieving statistical significance. Indeed, age, sex and obesity have already clearly been identified as risk factors for PE (40). Interestingly, the adjusted ORs of hypertension were less than 1.0, implying that hypertension appeared to be a protective factor for PE, which seems controversial with clinical practice. Consistent with this finding, Hu et al. (41) performed a summary-level Mendelian randomization analysis by extracting data from public and large-scale genome-wide association studies, and found that per SD increase of systolic blood pressure (SBP) could decrease the risk of PE by 1% (95% CI: 0.98–1.00, *P* = 0.003), and the presence of hypertension was associated with a lower risk of PE, although statistical significance was not reached (OR = 0.69, 95% CI: 0.42–1.15, *P* = 0.16). Another meta-analysis including 9 prospective studies also supported an inverse association between SBP and PE, and patients with hypertension had a lower risk of PE (hazard ratio = 0.93, 95% CI: 0.85–1.03) (42). The potential mechanisms behind the protective effect of hypertension are not well understood. It was likely that the increased medical attention after hypertension diagnosis may be a protective factor against PE (41). Further studies are needed to validate this finding.

Cancer has long been recognized as an important risk factor for PE (28, 40). In this study, patients with PE had a higher proportion of cancer than those without (20.54% vs. 14.42%), and univariate analysis showed that patients with cancer had a 1.535-fold increase in PE risk (*P* = 0.026). However, this relationship lost its independent significance in the multivariate analysis and sensitivity analysis (Model 1 and Model 2). When compared with patients without cancer, those with cancer were younger (65.26 vs. 70.32 years, *P* < 0.001), and less obesity (5.40% vs. 7.61%, *P* = 0.038). This may have contributed to the lack of statistical significance found in cancer.

Some potential limitations should be noted. First, this was a retrospective study, some important variables related to PE could not be accessed, including other risk factors (e.g., prior VTE, immobility, trauma), anticoagulation and antiplatelet use before hospital admission, and location of PE (central [main or lobar pulmonary artery branch] vs. peripheral [segmental or subsegmental branch]). Meanwhile, anticoagulants after LEDVT diagnosis were not obtained due to lack of detailed medication regimen in the database. Second, all patients were identified from our hospital, hence selection bias inevitably existed. Also, excluding a large number of patients with unilateral CUS examination from the analysis may have led to a selection bias. Despite the strengths of our study, the findings should be interpreted with some caution. Third, although the overall sample size was large, the sample size for PE were relatively small. For this reason, a further subdivision of anatomic distribution of LEDVT (left-distal, left-proximal, right-distal, right-proximal) could not be analyzed. Therefore, more studies are necessary to validate these associations.

In conclusion, patients with unilateral-proximal, bilateral-distal or bilateral-proximal are more likely to suffer from PE than those with unilateral-distal LEDVT.

## Data Availability

The raw data supporting the conclusions of this article will be made available by the authors, without undue reservation.

## Supplementary material

Supplementary Figure S1 Association between left, right LEDVT and pulmonary embolism risk using a multivariate sensitivity analysis by excluding patients with missing data.

Supplementary Figure S2 Receiver operating characteristic (ROC) curve of age for the detection of pulmonary embolism.
