# Supplementary material for: Anatomic distribution of lower extremity deep venous thrombosis is associated with an increased risk of pulmonary embolism: A 10-year retrospective analysis
Source: Front Cardiovasc Med. 2023 Mar 22;10:1154875. doi: 10.3389/fcvm.2023.1154875 (PMC10073460; doi:10.3389/fcvm.2023.1154875)
Supplement: Supplementary file 1 [file Table1.docx]

**Table S1 Univariate and multivariate logistic regression analyses of factors (including right LEDVT) associated with pulmonary embolism (Model 1).**

| **Variables** | **Univariate** | | **Multivariate** | |
| --- | --- | --- | --- | --- |
|  | **OR (95% *CI*)** | ***P* value** | **OR (95% *CI*)** | ***P* value** |
| Age (≥ 75 vs < 75 years) | 1.673 (1.205-2.323) | 0.002 | 1.427 (1.010-2.016) | 0.044 |
| Sex (men vs women) | 1.306 (0.966-1.765) | 0.083 | 1.284 (0.940-1.755) | 0.116 |
| Obesity (yes vs no) | 1.688 (1.012-2.817) | 0.045 | 1.856 (1.094-3.150) | 0.022 |
| Hypertension (yes vs no) | 0.600 (0.430-0.838) | 0.003 | 0.700 (0.493-0.995) | 0.047 |
| Renal insufficiency (yes vs no) | 0.481 (0.233-0.993) | 0.048 | 0.531 (0.253-1.115) | 0.095 |
| Cancer (yes vs no) | 1.535 (1.053-2.235) | 0.026 | 1.199 (0.808-1.781) | 0.367 |
| Thrombus sidedness (vs left) |  |  |  |  |
| Right LEDVT | 1.439 (0.932-2.221) | 0.100 | 1.544 (0.995-2.394) | 0.053 |
| Bilateral LEDVT | 2.830 (1.933-4.144) | <0.001 | 3.038 (2.060-4.480) | <0.001 |
| Thrombus location (vs distal) |  |  |  |  |
| Proximal LEDVT | 1.745 (1.282-2.377) | <0.001 | 1.554 (1.121-2.153) | 0.008 |

OR, odds ratio; CI, confidence interval; LEDVT, lower extremity deep venous thrombosis.
