# Supplementary material for: Anatomic distribution of lower extremity deep venous thrombosis is associated with an increased risk of pulmonary embolism: A 10-year retrospective analysis
Source: Front Cardiovasc Med. 2023 Mar 22;10:1154875. doi: 10.3389/fcvm.2023.1154875 (PMC10073460; doi:10.3389/fcvm.2023.1154875)
Supplement: Supplementary file 2 [file Table2.docx]

**Table S2 Multicollinearity analysis with variance inflation factor for multivariate Model 1 and Model 2.**

| **Variables** | **Model 1** | | **Variables** | **Model 2** | |
| --- | --- | --- | --- | --- | --- |
|  | **Multivariate analysis** | **Sensitivity analysis** |  | **Multivariate analysis** | **Sensitivity analysis** |
| Age | 1.086 | 1.093 | Age | 1.087 | 1.093 |
| Sex | 1.023 | 1.018 | Sex | 1.027 | 1.023 |
| Obesity | 1.762 | 1.016 | Obesity | 1.763 | 1.016 |
| Hypertension | 1.092 | 1.104 | Hypertension | 1.093 | 1.104 |
| Renal insufficiency | 1.049 | 1.048 | Renal insufficiency | 1.049 | 1.048 |
| Cancer | 1.045 | 1.044 | Cancer | 1.051 | 1.049 |
| Bilateral LEDVT | 1.014 | 1.013 | Unilateral-proximal LEDVT | 1.786 | 1.939 |
| Proximal LEDVT | 1.061 | 1.021 | Bilateral-proximal LEDVT | 2.188 | 2.443 |
|  |  |  | Bilateral-distal LEDVT | 1.674 | 1.801 |

LEDVT, lower extremity deep venous thrombosis.
