# Supplementary figures and images for: Anatomic distribution of lower extremity deep venous thrombosis is associated with an increased risk of pulmonary embolism: A 10-year retrospective analysis
Source: Front Cardiovasc Med. 2023 Mar 22;10:1154875. doi: 10.3389/fcvm.2023.1154875 (PMC10073460; doi:10.3389/fcvm.2023.1154875)

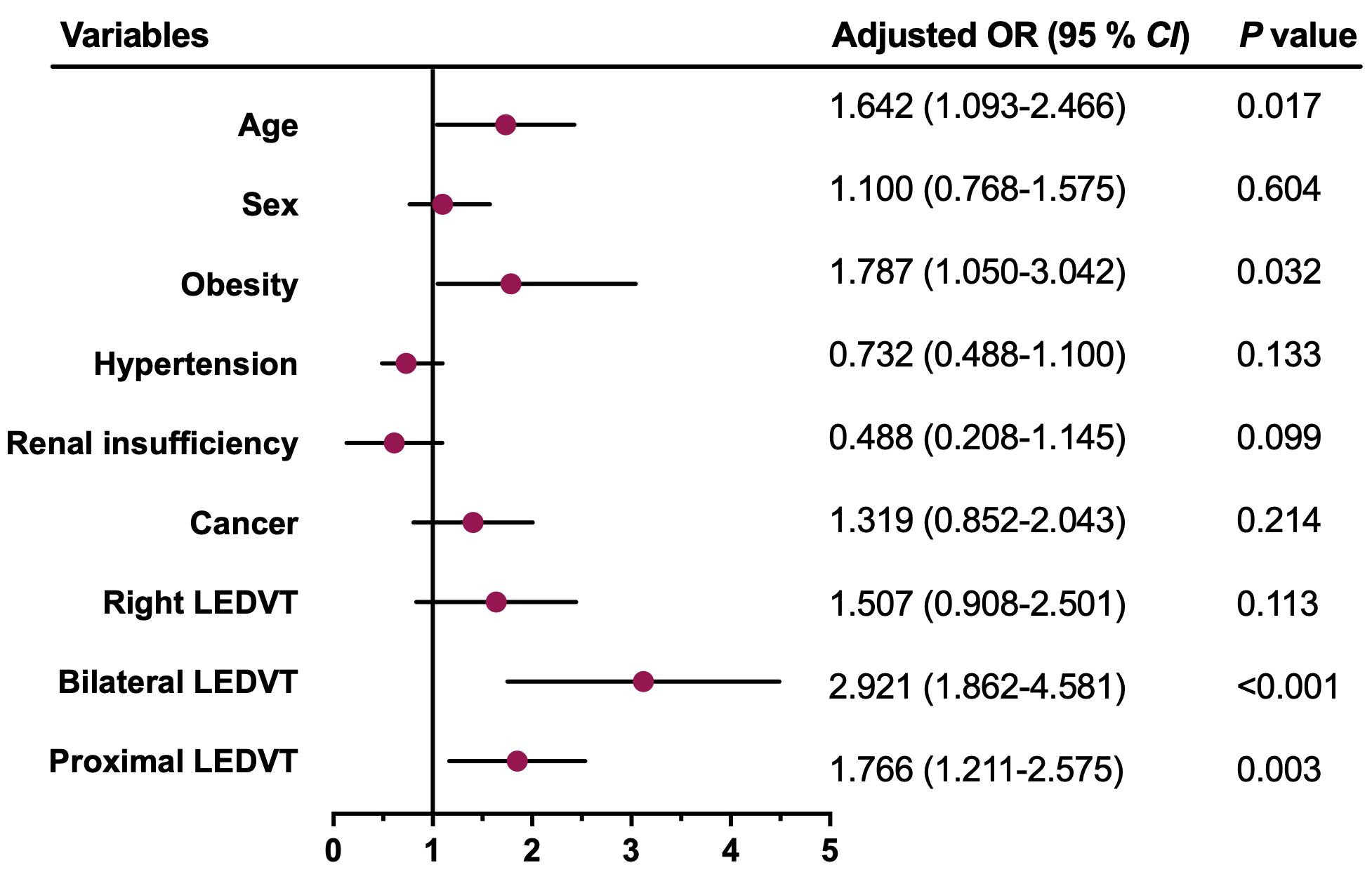

Supplement: Supplementary Figure S1 — Association between left, right LEDVT and pulmonary embolism risk using a multivariate sensitivity analysis by excluding patients with missing data. [file Image1.jpeg]

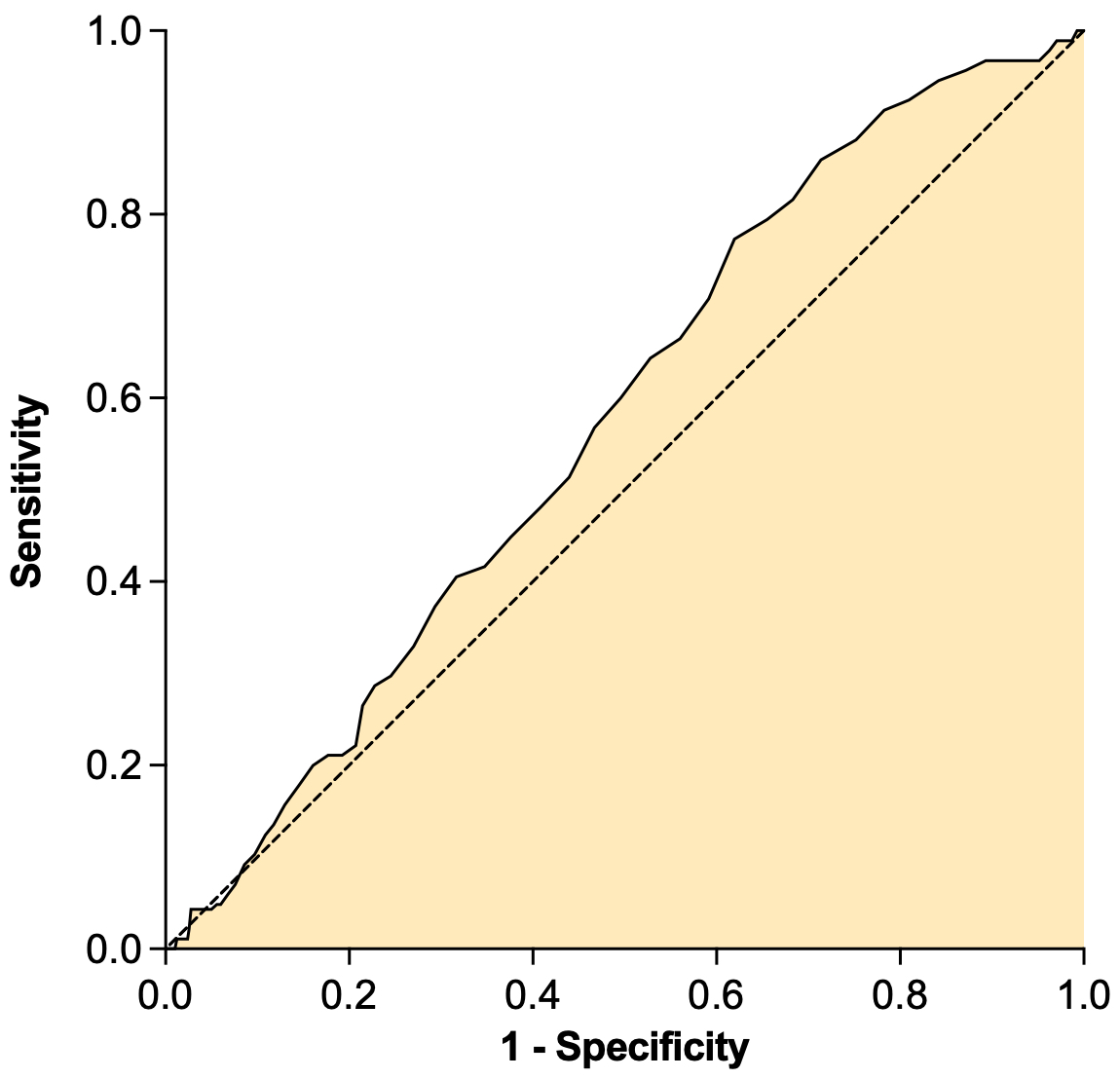

Supplement: Supplementary Figure S2 — Receiver operating characteristic (ROC) curve of age for the detection of pulmonary embolism. [file Image2.jpeg]
